# Supplementary material for: Advancing poultry health: A meta-analysis of epitope-based and peptide-based vaccines against Avian Pathogenic E. coli with machine learning insights
Source: PLoS One. 2026 May 27;21(5):e0349094. doi: 10.1371/journal.pone.0349094 (PMC13215497; doi:10.1371/journal.pone.0349094)
Supplement: S3 File — Statistical output includes pooled effect sizes, forest plots, heterogeneity measures (I2), and model estimates for morbidity prevention. (HTML) [file pone.0349094.s003.html]

Results


# Results

# Meta-Analysis

Effect Sizes, Sampling Variances or Standard Errors, and Study Label fields must be populated to run analysis

| Random-Effects Model | | | | | | | | | | | | | |
| --- | --- | --- | --- | --- | --- | --- | --- | --- | --- | --- | --- | --- | --- |
|  | | Estimate | | se | | Z | | p | | CI Lower Bound | | CI Upper Bound | |
| . |  | . |  | . |  | . |  | . |  | . |  | . |  |
| . |  | . |  | . |  | . |  | . |  | . |  | . |  |
|  |  |  |  |  |  |  |  |  |  |  |  |  |  |
| --- | --- | --- | --- | --- | --- | --- | --- | --- | --- | --- | --- | --- | --- |
|  | | | | | | | | | | | | | |
| [3] | | | | | | | | | | | | | |

| Heterogeneity Statistics | | | | | | | | | | | | | | | |
| --- | --- | --- | --- | --- | --- | --- | --- | --- | --- | --- | --- | --- | --- | --- | --- |
| Tau | | Tau² | | I² | | H² | | R² | | df | | Q | | p | |
| . |  | . |  | . |  | . |  | . |  | . |  | . |  | . |  |
|  |  |  |  |  |  |  |  |  |  |  |  |  |  |  |  |
| --- | --- | --- | --- | --- | --- | --- | --- | --- | --- | --- | --- | --- | --- | --- | --- |
|  | | | | | | | | | | | | | | | |
|  | | | | | | | | | | | | | | | |

| Model Fit Statistics and Information Criteria | | | | | | | | | | | |
| --- | --- | --- | --- | --- | --- | --- | --- | --- | --- | --- | --- |
|  | | log-likelihood | | Deviance | | AIC | | BIC | | AICc | |
| . |  | . |  | . |  | . |  | . |  | . |  |
| . |  | . |  | . |  | . |  | . |  | . |  |
|  |  |  |  |  |  |  |  |  |  |  |  |
| --- | --- | --- | --- | --- | --- | --- | --- | --- | --- | --- | --- |
|  | | | | | | | | | | | |
|  | | | | | | | | | | | |

## Forest Plot

[3]

|  | | | | | |
| --- | --- | --- | --- | --- | --- |
| Test Name | | value | | p | |
| . |  | . |  | . |  |
| . |  | . |  | . |  |
| . |  | . |  | . |  |
|  |  |  |  |  |  |
| --- | --- | --- | --- | --- | --- |
|  | | | | | |
|  | | | | | |

## Funnel Plot

[3]

| Two One-Sided Tests Equivalence Testing | | | | | | | | | | | | | | | |
| --- | --- | --- | --- | --- | --- | --- | --- | --- | --- | --- | --- | --- | --- | --- | --- |
| Z-Value Lower Bound | | P-Value Lower Bound | | Z-Value Upper Bound | | P-Value Upper Bound | | LL\_CI\_TOST | | UL\_CI\_TOST | | LL\_CI\_ZTEST | | UL\_CI\_ZTEST | |
| . |  | . |  | . |  | . |  | . |  | . |  | . |  | . |  |
|  |  |  |  |  |  |  |  |  |  |  |  |  |  |  |  |
| --- | --- | --- | --- | --- | --- | --- | --- | --- | --- | --- | --- | --- | --- | --- | --- |
|  | | | | | | | | | | | | | | | |
| [4] | | | | | | | | | | | | | | | |

## Two One-Sided Tests Equivalence Testing: Text Summary

## Equivalence Test Plot

[4]

## Outlier and Influential Case Diagnostics

### Externally Standardized Residual

### DFFITS Values

### Cook's Distances

### Covariance Ratios

### Leave-one-out Tau Estimates

### Leave-one-out (residual) Heterogeneity Test Statistics

### Hat Values

### Weights

### Q-Q Plot

# Meta-Analysis

| Random-Effects Model (k = 8) | | | | | | | | | | | | | |
| --- | --- | --- | --- | --- | --- | --- | --- | --- | --- | --- | --- | --- | --- |
|  | | Estimate | | se | | Z | | p | | CI Lower Bound | | CI Upper Bound | |
| Intercept |  | 1.49 |  | 0.0969 |  | 15.4 |  | < .001 |  | 1.305 |  | 1.684 |  |
|  |  | . |  | . |  | . |  | . |  | . |  | . |  |
|  |  |  |  |  |  |  |  |  |  |  |  |  |  |
| --- | --- | --- | --- | --- | --- | --- | --- | --- | --- | --- | --- | --- | --- |
| Note. Tau² Estimator: Restricted Maximum-Likelihood | | | | | | | | | | | | | |
|  | | | | | | | | | | | | | |
| [3] | | | | | | | | | | | | | |

| Heterogeneity Statistics | | | | | | | | | | | | | | | |
| --- | --- | --- | --- | --- | --- | --- | --- | --- | --- | --- | --- | --- | --- | --- | --- |
| Tau | | Tau² | | I² | | H² | | R² | | df | | Q | | p | |
| 0.067 |  | 0.0044 (SE= 0.0391 ) |  | 5.85% |  | 1.062 |  | . |  | 7.000 |  | 8.736 |  | 0.272 |  |
|  |  |  |  |  |  |  |  |  |  |  |  |  |  |  |  |
| --- | --- | --- | --- | --- | --- | --- | --- | --- | --- | --- | --- | --- | --- | --- | --- |
|  | | | | | | | | | | | | | | | |
|  | | | | | | | | | | | | | | | |

## Forest Plot

[3]


| Publication Bias Assessment | | | | | |
| --- | --- | --- | --- | --- | --- |
| Test Name | | value | | p | |
| Fail-Safe N |  | 726.000 |  | < .001 |  |
| Kendalls Tau |  | -0.071 |  | 0.905 |  |
| Egger's Regression |  | 0.119 |  | 0.905 |  |
|  |  |  |  |  |  |
| --- | --- | --- | --- | --- | --- |
| Note. Fail-safe N Calculation Using the Rosenthal Approach | | | | | |
|  | | | | | |
|  | | | | | |

## Funnel Plot

[3]


| Two One-Sided Tests Equivalence Testing | | | | | | | | | | | | | | | |
| --- | --- | --- | --- | --- | --- | --- | --- | --- | --- | --- | --- | --- | --- | --- | --- |
| Z-Value Lower Bound | | P-Value Lower Bound | | Z-Value Upper Bound | | P-Value Upper Bound | | LL\_CI\_TOST | | UL\_CI\_TOST | | LL\_CI\_ZTEST | | UL\_CI\_ZTEST | |
| 20.587 |  | < .001 |  | 10.266 |  | 1.000 |  | 1.335 |  | 1.654 |  | 1.305 |  | 1.684 |  |
|  |  |  |  |  |  |  |  |  |  |  |  |  |  |  |  |
| --- | --- | --- | --- | --- | --- | --- | --- | --- | --- | --- | --- | --- | --- | --- | --- |
|  | | | | | | | | | | | | | | | |
| [4] | | | | | | | | | | | | | | | |

## Two One-Sided Tests Equivalence Testing: Text Summary

```
The equivalence test was non-significant, Z = 10.266, p = 1.000, given equivalence bounds of -0.500 and 0.500 and an alpha of 0.05.
The null hypothesis test was significant, Z = 15.426, p = 0.0000000000000000000000000000000000000000000000000000109, given an alpha of 0.05.
NA
```

## Equivalence Test Plot

[4]


# Meta-Analysis

| Random-Effects Model (k = 8) | | | | | | | | | | | | | |
| --- | --- | --- | --- | --- | --- | --- | --- | --- | --- | --- | --- | --- | --- |
|  | | Estimate | | se | | Z | | p | | CI Lower Bound | | CI Upper Bound | |
| Intercept |  | 1.50 |  | 0.116 |  | 12.8 |  | < .001 |  | 1.267 |  | 1.724 |  |
|  |  | . |  | . |  | . |  | . |  | . |  | . |  |
|  |  |  |  |  |  |  |  |  |  |  |  |  |  |
| --- | --- | --- | --- | --- | --- | --- | --- | --- | --- | --- | --- | --- | --- |
| Note. Tau² Estimator: Restricted Maximum-Likelihood | | | | | | | | | | | | | |
|  | | | | | | | | | | | | | |
| [3] | | | | | | | | | | | | | |

| Heterogeneity Statistics | | | | | | | | | | | | | | | |
| --- | --- | --- | --- | --- | --- | --- | --- | --- | --- | --- | --- | --- | --- | --- | --- |
| Tau | | Tau² | | I² | | H² | | R² | | df | | Q | | p | |
| 0.318 |  | 0.1013 (SE= 0.0579 ) |  | 95.56% |  | 22.499 |  | . |  | 7.000 |  | 102.669 |  | < .001 |  |
|  |  |  |  |  |  |  |  |  |  |  |  |  |  |  |  |
| --- | --- | --- | --- | --- | --- | --- | --- | --- | --- | --- | --- | --- | --- | --- | --- |
|  | | | | | | | | | | | | | | | |
|  | | | | | | | | | | | | | | | |

## Forest Plot

[3]


| Publication Bias Assessment | | | | | |
| --- | --- | --- | --- | --- | --- |
| Test Name | | value | | p | |
| Fail-Safe N |  | 10689.000 |  | < .001 |  |
| Kendalls Tau |  | -0.071 |  | 0.905 |  |
| Egger's Regression |  | 0.618 |  | 0.537 |  |
|  |  |  |  |  |  |
| --- | --- | --- | --- | --- | --- |
| Note. Fail-safe N Calculation Using the Rosenthal Approach | | | | | |
|  | | | | | |
|  | | | | | |

## Funnel Plot

[3]


| Two One-Sided Tests Equivalence Testing | | | | | | | | | | | | | | | |
| --- | --- | --- | --- | --- | --- | --- | --- | --- | --- | --- | --- | --- | --- | --- | --- |
| Z-Value Lower Bound | | P-Value Lower Bound | | Z-Value Upper Bound | | P-Value Upper Bound | | LL\_CI\_TOST | | UL\_CI\_TOST | | LL\_CI\_ZTEST | | UL\_CI\_ZTEST | |
| 17.140 |  | < .001 |  | 8.550 |  | 1.000 |  | 1.304 |  | 1.687 |  | 1.267 |  | 1.724 |  |
|  |  |  |  |  |  |  |  |  |  |  |  |  |  |  |  |
| --- | --- | --- | --- | --- | --- | --- | --- | --- | --- | --- | --- | --- | --- | --- | --- |
|  | | | | | | | | | | | | | | | |
| [4] | | | | | | | | | | | | | | | |

## Two One-Sided Tests Equivalence Testing: Text Summary

```
The equivalence test was non-significant, Z = 8.550, p = 1.000, given equivalence bounds of -0.500 and 0.500 and an alpha of 0.05.
The null hypothesis test was significant, Z = 12.845, p = 0.0000000000000000000000000000000000000917, given an alpha of 0.05.
NA
```

## Equivalence Test Plot

[4]


# Meta-Analysis

Effect Sizes, Sampling Variances or Standard Errors, and Study Label fields must be populated to run analysis

| Random-Effects Model | | | | | | | | | | | | | |
| --- | --- | --- | --- | --- | --- | --- | --- | --- | --- | --- | --- | --- | --- |
|  | | Estimate | | se | | Z | | p | | CI Lower Bound | | CI Upper Bound | |
| . |  | . |  | . |  | . |  | . |  | . |  | . |  |
| . |  | . |  | . |  | . |  | . |  | . |  | . |  |
|  |  |  |  |  |  |  |  |  |  |  |  |  |  |
| --- | --- | --- | --- | --- | --- | --- | --- | --- | --- | --- | --- | --- | --- |
|  | | | | | | | | | | | | | |
| [3] | | | | | | | | | | | | | |

| Heterogeneity Statistics | | | | | | | | | | | | | | | |
| --- | --- | --- | --- | --- | --- | --- | --- | --- | --- | --- | --- | --- | --- | --- | --- |
| Tau | | Tau² | | I² | | H² | | R² | | df | | Q | | p | |
| . |  | . |  | . |  | . |  | . |  | . |  | . |  | . |  |
|  |  |  |  |  |  |  |  |  |  |  |  |  |  |  |  |
| --- | --- | --- | --- | --- | --- | --- | --- | --- | --- | --- | --- | --- | --- | --- | --- |
|  | | | | | | | | | | | | | | | |
|  | | | | | | | | | | | | | | | |

| Model Fit Statistics and Information Criteria | | | | | | | | | | | |
| --- | --- | --- | --- | --- | --- | --- | --- | --- | --- | --- | --- |
|  | | log-likelihood | | Deviance | | AIC | | BIC | | AICc | |
| . |  | . |  | . |  | . |  | . |  | . |  |
| . |  | . |  | . |  | . |  | . |  | . |  |
|  |  |  |  |  |  |  |  |  |  |  |  |
| --- | --- | --- | --- | --- | --- | --- | --- | --- | --- | --- | --- |
|  | | | | | | | | | | | |
|  | | | | | | | | | | | |

## Forest Plot

[3]

|  | | | | | |
| --- | --- | --- | --- | --- | --- |
| Test Name | | value | | p | |
| . |  | . |  | . |  |
| . |  | . |  | . |  |
| . |  | . |  | . |  |
|  |  |  |  |  |  |
| --- | --- | --- | --- | --- | --- |
|  | | | | | |
|  | | | | | |

## Funnel Plot

[3]

| Two One-Sided Tests Equivalence Testing | | | | | | | | | | | | | | | |
| --- | --- | --- | --- | --- | --- | --- | --- | --- | --- | --- | --- | --- | --- | --- | --- |
| Z-Value Lower Bound | | P-Value Lower Bound | | Z-Value Upper Bound | | P-Value Upper Bound | | LL\_CI\_TOST | | UL\_CI\_TOST | | LL\_CI\_ZTEST | | UL\_CI\_ZTEST | |
| . |  | . |  | . |  | . |  | . |  | . |  | . |  | . |  |
|  |  |  |  |  |  |  |  |  |  |  |  |  |  |  |  |
| --- | --- | --- | --- | --- | --- | --- | --- | --- | --- | --- | --- | --- | --- | --- | --- |
|  | | | | | | | | | | | | | | | |
| [4] | | | | | | | | | | | | | | | |

## Two One-Sided Tests Equivalence Testing: Text Summary

## Equivalence Test Plot

[4]

## Outlier and Influential Case Diagnostics

### Externally Standardized Residual

### DFFITS Values

### Cook's Distances

### Covariance Ratios

### Leave-one-out Tau Estimates

### Leave-one-out (residual) Heterogeneity Test Statistics

### Hat Values

### Weights

### Q-Q Plot

# Meta-Analysis

| Random-Effects Model (k = 8) | | | | | | | | | | | | | |
| --- | --- | --- | --- | --- | --- | --- | --- | --- | --- | --- | --- | --- | --- |
|  | | Estimate | | se | | Z | | p | | CI Lower Bound | | CI Upper Bound | |
| Intercept |  | 1.49 |  | 0.0969 |  | 15.4 |  | < .001 |  | 1.305 |  | 1.684 |  |
|  |  | . |  | . |  | . |  | . |  | . |  | . |  |
|  |  |  |  |  |  |  |  |  |  |  |  |  |  |
| --- | --- | --- | --- | --- | --- | --- | --- | --- | --- | --- | --- | --- | --- |
| Note. Tau² Estimator: Restricted Maximum-Likelihood | | | | | | | | | | | | | |
|  | | | | | | | | | | | | | |
| [3] | | | | | | | | | | | | | |

| Heterogeneity Statistics | | | | | | | | | | | | | | | |
| --- | --- | --- | --- | --- | --- | --- | --- | --- | --- | --- | --- | --- | --- | --- | --- |
| Tau | | Tau² | | I² | | H² | | R² | | df | | Q | | p | |
| 0.067 |  | 0.0044 (SE= 0.0391 ) |  | 5.85% |  | 1.062 |  | . |  | 7.000 |  | 8.736 |  | 0.272 |  |
|  |  |  |  |  |  |  |  |  |  |  |  |  |  |  |  |
| --- | --- | --- | --- | --- | --- | --- | --- | --- | --- | --- | --- | --- | --- | --- | --- |
|  | | | | | | | | | | | | | | | |
|  | | | | | | | | | | | | | | | |

## Forest Plot

[3]


| Publication Bias Assessment | | | | | |
| --- | --- | --- | --- | --- | --- |
| Test Name | | value | | p | |
| Fail-Safe N |  | 726.000 |  | < .001 |  |
| Kendalls Tau |  | -0.071 |  | 0.905 |  |
| Egger's Regression |  | 0.119 |  | 0.905 |  |
|  |  |  |  |  |  |
| --- | --- | --- | --- | --- | --- |
| Note. Fail-safe N Calculation Using the Rosenthal Approach | | | | | |
|  | | | | | |
|  | | | | | |

## Funnel Plot

[3]


| Two One-Sided Tests Equivalence Testing | | | | | | | | | | | | | | | |
| --- | --- | --- | --- | --- | --- | --- | --- | --- | --- | --- | --- | --- | --- | --- | --- |
| Z-Value Lower Bound | | P-Value Lower Bound | | Z-Value Upper Bound | | P-Value Upper Bound | | LL\_CI\_TOST | | UL\_CI\_TOST | | LL\_CI\_ZTEST | | UL\_CI\_ZTEST | |
| 20.587 |  | < .001 |  | 10.266 |  | 1.000 |  | 1.335 |  | 1.654 |  | 1.305 |  | 1.684 |  |
|  |  |  |  |  |  |  |  |  |  |  |  |  |  |  |  |
| --- | --- | --- | --- | --- | --- | --- | --- | --- | --- | --- | --- | --- | --- | --- | --- |
|  | | | | | | | | | | | | | | | |
| [4] | | | | | | | | | | | | | | | |

## Two One-Sided Tests Equivalence Testing: Text Summary

```
The equivalence test was non-significant, Z = 10.266, p = 1.000, given equivalence bounds of -0.500 and 0.500 and an alpha of 0.05.
The null hypothesis test was significant, Z = 15.426, p = 0.0000000000000000000000000000000000000000000000000000109, given an alpha of 0.05.
NA
```

## Equivalence Test Plot

[4]


# References

[1]
The jamovi project (2023). *jamovi*. (Version 2.4) [Computer Software]. Retrieved from https://www.jamovi.org.

[2]
R Core Team (2022). *R: A Language and environment for statistical computing*. (Version 4.1) [Computer software]. Retrieved from https://cran.r-project.org. (R packages retrieved from CRAN snapshot 2023-04-07).

[3]
Viechtbauer, W. (2010). Conducting meta-analyses in R with the metafor package. *Journal of Statistical Software. link, 36*, 1-48.

[4]
Lakens, D. (2017). Equivalence tests: A practical primer for t-tests, correlations, and meta-analyses. *Social Psychological and Personality Science. link, 1*, 1-8.
