# Supplementary figures and images for: Advancing poultry health: A meta-analysis of epitope-based and peptide-based vaccines against Avian Pathogenic E. coli with machine learning insights
Source: PLoS One. 2026 May 27;21(5):e0349094. doi: 10.1371/journal.pone.0349094 (PMC13215497; doi:10.1371/journal.pone.0349094)

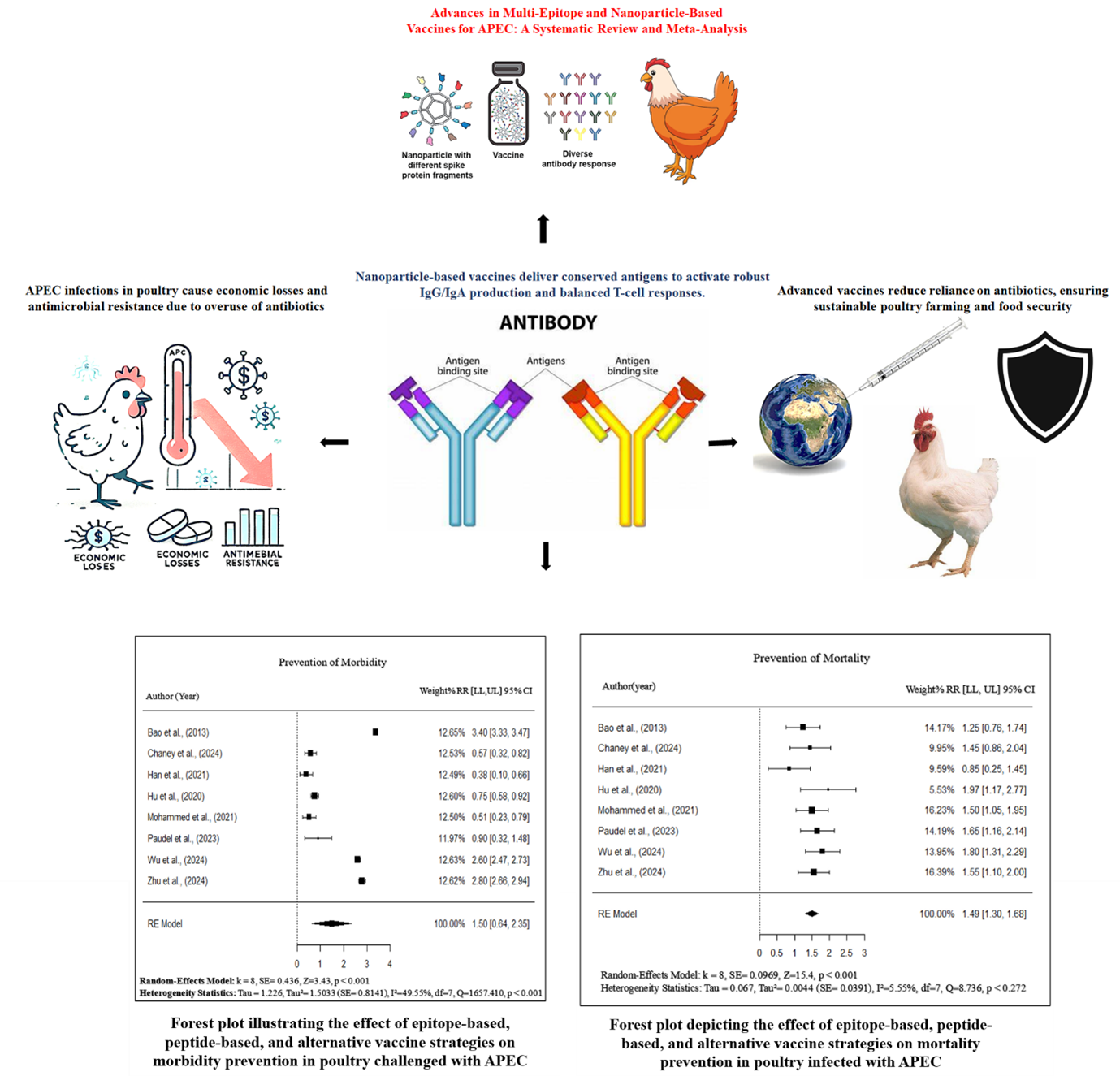

Supplement: S1 Fig — (PNG) [file pone.0349094.s005.png]
