# Supplementary material for: Advancing poultry health: A meta-analysis of epitope-based and peptide-based vaccines against Avian Pathogenic E. coli with machine learning insights
Source: PLoS One. 2026 May 27;21(5):e0349094. doi: 10.1371/journal.pone.0349094 (PMC13215497; doi:10.1371/journal.pone.0349094)
Supplement: Attachment — Submitted filename: Peer Review.PONE-D-25-45349.docx [file pone.0349094.s006.docx]

Dear Editor,

I appreciate the opportunity to review the manuscript entitled “***Advancing Poultry Health: A Meta-Analysis of Epitope-Based and Peptide-Based Vaccines Against Avian Pathogenic E. coli with Machine Learning Insights***” (Manuscript ID: PONE-D-25-45349).

Please find attached my completed review report. I have provided comments in a constructive, respectful, and objective manner, aiming to support both the authors and the editorial process. I hope the remarks will be helpful in evaluating the scientific merit and clarity of the manuscript.

Thank you for considering my review.

Sincerely,

**Dr. Alemeh Hosseinian**

**Peer review**

The manuscript addresses an important topic concerning epitope- and peptide-based vaccines against APEC and provides a timely synthesis that integrates machine-learning applications. The subject is relevant to poultry health, AMR reduction, and vaccine innovation. The manuscript is generally well-structured and clearly written; however, several areas would benefit from clarification or methodological strengthening to enhance scientific rigor and transparency.

________________________________________

**Major Comments**

1.	Scope and Positioning

While the review integrates ML as a major theme, the included studies do not uniformly apply ML. Clarifying the precise contribution of ML in the synthesis would improve conceptual coherence.

2.	Methodology Transparency

The PRISMA methods are appropriately mentioned, but more detail is needed regarding search strings, inclusion/exclusion procedures, and the handling of AI-only narrative papers.

3.	Meta-Analysis Details

Effect size values (RR > 1) appear to indicate increased risk rather than reduction unless inverted—clarification of directionality is recommended. Additionally, missing numeric data for several forest plots limits reproducibility.

4.	Study Heterogeneity

Although heterogeneity is reported as low, differences in vaccine platforms, serotypes, species, and outcome measures should be acknowledged more explicitly.

5.	Machine Learning Section

The ML component is promising but mostly descriptive. Strengthening this part with more concrete methodological criteria or limitations would improve the scientific contribution.

________________________________________

**Comments**

**Title & Abstract**

Clear and informative. The abstract would benefit from numerical context on included studies and a more cautious interpretation of ML findings.

**Introduction**

Well-written and comprehensive. A slightly tighter focus on the rationale for combining meta-analysis with ML discussion would increase coherence.

**Materials & Methods**

The PRISMA-based approach is appropriate; however, clarification on risk-of-bias scoring, data extraction consistency, and management of narrative ML-related information is recommended.

**Results**

Results are clearly structured. Nevertheless, the reporting of effect sizes, heterogeneity, and subgroup analyses should be made more transparent, especially regarding exact study counts and numerical inputs.

**Discussion**

The discussion provides valuable synthesis but could benefit from more balanced coverage of limitations, including dataset variability, model validation challenges, and real-world applicability of ML-based predictions.

**Conclusion**

Concise and aligned with findings. A more moderate tone regarding the transformative potential of ML would strengthen scientific neutrality.

**Tables & Figures**

Tables are informative. Forest plots and the PRISMA diagram should include complete numeric labels to improve reproducibility.

**References**

Comprehensive and up-to-date. Minor formatting inconsistencies can be corrected during editing.

________________________________________

**Recommendation**

**Major Revision**

The manuscript presents a valuable contribution; however, several methodological clarifications and refinements to the ML integration are needed before it can be considered for publication.
