# Supplementary material for: Advancing poultry health: A meta-analysis of epitope-based and peptide-based vaccines against Avian Pathogenic E. coli with machine learning insights
Source: PLoS One. 2026 May 27;21(5):e0349094. doi: 10.1371/journal.pone.0349094 (PMC13215497; doi:10.1371/journal.pone.0349094)
Supplement: Attachment — Submitted filename: Response to Reviewer new.docx [file pone.0349094.s007.docx]

Dear Editor,

We would like to thank staff of PlosOne and the reviewers for careful and thorough analysis of the manuscript. We found the feedback of your team and reviewers very constructive. We tried to be responsive to your concerns and want to extend our appreciation for taking the time and effort necessary to provide such insightful guidance.

Your comments and those of the reviewers were highly insightful and enabled us to greatly improve the quality of our research paper. Please find attached file, a revised version of our manuscript entitled “***Advancing Poultry Health: A Meta-Analysis of Epitope-Based and Peptide-Based Vaccines Against Avian Pathogenic E. coli with Machine Learning Insights***” (Manuscript ID: PONE-D-25-45349)**.**

We hope that these revisions improve the overall quality of our write-up such that you and the reviewers now deem it worthy of publication in the PlosOne journal. Next, we offer brief responses to your comments as well as those of the reviewers.

The manuscript has been improved by considering your and reviewer’s valuable suggestions. We hope that you and the referees will find our responses satisfactory, and we are willing to include future suggestions.

Below, we respond to the comments by the referees, and indicate where relevant changes have been made. We look forward to working with you and the referees to move this manuscript closer to publication in the PlosOne journal.

In case of further queries, we will be happy to provide further details and look forward to your response.

Sincerely,

Amjad Ali, PhD

**Peer review**

The manuscript addresses an important topic concerning epitope- and peptide-based vaccines against APEC and provides a timely synthesis that integrates machine-learning applications. The subject is relevant to poultry health, AMR reduction, and vaccine innovation. The manuscript is generally well-structured and clearly written; however, several areas would benefit from clarification or methodological strengthening to enhance scientific rigor and transparency.

**Response:** We thank reviewers for taking time and constructively review our manuscript.

________________________________________

**Major Comments**

*1.	Scope and Positioning*

*While the review integrates ML as a major theme, the included studies do not uniformly apply ML. Clarifying the precise contribution of ML in the synthesis would improve conceptual coherence.*

**Response**: We greatly appreciate the reviewer’s feedback, ML synthesis and impact in this manuscript are added at line # 227 to 237. Kindly review.

*2.	Methodology Transparency*

*The PRISMA methods are appropriately mentioned, but more detail is needed regarding search strings, inclusion/exclusion procedures, and the handling of AI-only narrative papers.*

**Response:** We are thankful for your valuable suggestion. The search strings are updated in line # 178 to 185, inclusion/exclusion criteria is added in line # 147 to 168, and handling of AI only narrative papers in line # 227 to 237.

*3.	Meta-Analysis Details*

*Effect size values (RR > 1) appear to indicate increased risk rather than reduction unless inverted—clarification of directionality is recommended. Additionally, missing numeric data for several forest plots limits reproducibility.*

**Response**: The wording has been inverted to prevention of mortality and morbidity to align interpretation with RR statistics. Kindly review meta-plots and figures with interpretation at line # 303 to 345.

*4.	Study Heterogeneity*

*Although heterogeneity is reported as low, differences in vaccine platforms, serotypes, species, and outcome measures should be acknowledged more explicitly.*

**Response:** We are thankful for your valuable suggestion. The subgroup analysis is presented in table 4 and interpretation in line # 329 to 429. Kindly review.

*5.	Machine Learning Section*

*The ML component is promising but mostly descriptive. Strengthening this part with more concrete methodological criteria or limitations would improve the scientific contribution.*

**Response:** We greatly appreciate the reviewer’s feedback. The methodological criteria is added in line # 227 to 237 and limitations of current AI/ML is already written and acknowledged in 518 to 528, but it is updated as per your suggestion in line # 529 to 536.

________________________________________

**Comments**

***Title & Abstract***

*Clear and informative. The abstract would benefit from numerical context on included studies and a more cautious interpretation of ML findings.*

**Response:** We thank the reviewer’s feedback. The revision has been made in the manuscript at line # 29 to 35.

***Introduction***

*Well-written and comprehensive. A slightly tighter focus on the rationale for combining meta-analysis with ML discussion would increase coherence.*

**Response:** The reviewer’s concern has been addressed in revised manuscript, the revision has been made on line # 87 to 90, 94 to 102, 129 to 133.

***Materials & Methods***

*The PRISMA-based approach is appropriate; however, clarification on risk-of-bias scoring, data extraction consistency, and management of narrative ML-related information is recommended.*

**Response:** The PRISMA scoring is added in methodology line # 220 to 225 and reported in result section 293 to 301. The plots have been added as figure 2,3 updated figures. Secondly, data extraction line # 202 to 214 and ML management of narrative is explained in data synthesis line 227 to 237. The below given references have been added in manuscript and highlighted in yellow.

- Higgins JP, Savović J, Page MJ, Elbers RG, Sterne JAC. Chapter 8: Assessing risk of bias in a randomized trial [last updated October 2019]. Available from: [Chapter 8: Assessing risk of bias in a randomized trial | Cochrane](https://www.cochrane.org/authors/handbooks-and-manuals/handbook/current/chapter-08)
- Sterne JA, Hernán MA, Reeves BC, Savović J, Berkman ND, Viswanathan M, Henry D, Altman DG, Ansari MT, Boutron I, Carpenter JR. ROBINS-I: a tool for assessing risk of bias in non-randomised studies of interventions. bmj. 2016 Oct 12;355. <https://doi.org/10.1136/bmj.i4919>

***Results***

*Results are clearly structured. Nevertheless, the reporting of effect sizes, heterogeneity, and subgroup analyses should be made more transparent, especially regarding exact study counts and numerical inputs.*

**Response:** Respected reviewer, the meta-plots, subgroup analysis statistics and counts in PRISMA has been consistently reported and updated as per your suggestion. Kindly review the figure 1 to 7.

***Discussion***

*The discussion provides valuable synthesis but could benefit from more balanced coverage of limitations, including dataset variability, model validation challenges, and real-world applicability of ML-based predictions.*

**Response:** Respected reviewer, the revision has been made. kindly review lines 529 to 536.

***Conclusion***

*Concise and aligned with findings. A more moderate tone regarding the transformative potential of ML would strengthen scientific neutrality.*

**Response:** We are thankful for your valuable suggestion, the conclusion section has been updated and in the revised manuscript on line number 558 to 584.

***Tables & Figures***

*Tables are informative. Forest plots and the PRISMA diagram should include complete numeric labels to improve reproducibility.*

**Response:** Respected reviewer, all new plots and figures have been updated and added. Kindly see figure 2-7.

***References***

*Comprehensive and up-to-date. Minor formatting inconsistencies can be corrected during editing.*

**Response:** Respected reviewer, we have made improvements in the formatting. However, if there is further improvements.

**Rebuttal Letter**

**Reviewer #1:**

*The reviewer's comments are well-noted. Many similar topics exist in review articles on this subject. Therefore, the meta-analysis should emphasize which specific findings it contributes. The article seems suitable for publication regardless.*

**Response:** We thank reviewers for taking time and constructively review our manuscript.

*Please consider the following points for revision:*

*1. The manuscript provides a weak explanation of the fundamental definition of machine learning (ML) and its application in the study, particularly within the methods section. The authors mention using ML for the "Study Selection Process." However, the legality of this application for selection requires clarification. Using third-party application programming interfaces is generally acceptable, unlike certain generative AI tools currently prevalent. The description lacks clarity on whether the team developed the ML process internally, as it omits the specific algorithms used. The authors must confirm these details and remember that ML and AI have distinct definitions.*

**Response:** Respected reviewer, I have refined the methodology section as per your suggestion. The author’s intended meaning was that the ML role in vaccine development and designing among included studies. The statement has been omitted. The data extraction and literature search were performed as per PRISMA 2020 statement guidelines. However, the synthesis section has been updated by incorporating qualitative synthesis of the ML role (Line # 220 to 239).

*2. The authors must confirm and clarify the statement: "For the purposes of this review, 'machine learning' was defined as a data-driven approach in which an algorithm learns patterns from input data (e.g., supervised, unsupervised, or deep learning methods)." The description fails to mention the programming language or software tools used to build the data extraction process with ML.*

**Response:** The reviewer’s concern has been addressed in revised manuscript. We have omitted these sentences, and actual intended meaning was to present the definition of ML to synthesis evidence. The synthesis approach is now elaborated in line 220 to 230.

*3. The writing in the "Materials and Methods" section suggests possible use of AI for editing, leading to typographical errors like: "Each domain was assessed as "low risk," "high risk," or "unclear risk," " The authors should carefully review the entire section.*

**Response:** Respected Reviewer, the methodological quality assessment (risk of bias assessment) has been added Line # 213 to 218. Kindly review how it is conducted using Cochrane guidelines. The reporting is added in result section line # 285 to 292 along with figure 2,3.

*4. In this reviewer's opinion, the article presents a standard meta-analysis. The use of ML appears limited to automating selection and data extraction, while the core analysis techniques follow conventional meta-analysis approaches. The authors should confirm this point and adjust the title and "Materials and Methods" section to reflect accurately the role of ML and AI.*

**Response:** Respected reviewer, the ML role is identified using synthesis given in line #220 to 230. Yes, the core analysis is performed using conventional meta-analysis along with qualitative synthesis on ML role.

*5. The manuscript repeats the full explanation for abbreviations, such as "outer membrane vesicles (OMVs)," multiple times. The authors should review and correct this pattern for all abbreviations used.*

**Response:** The reviewer’s concern has been addressed in revised manuscript.

*6. Figure 1, the PRISMA-P flowchart, should specify the reasons for excluding records during the screening. Eliminating 219 articles due to "different objectives" suggests a significant weakness in the initial keyword strategy. A more precise keyword selection would likely have prevented such a large-scale exclusion for that reason. The authors must provide a better explanation for these exclusions.*

**Response:** Respected reviewer, the exact reasons for exclusion have been added now in Figure 1 (PRISMA Flowchart).

*7. The abstract's graphical representation mentions vaccine effectiveness results, but the manuscript contains no corresponding forest plots. The reported parameters are also surprisingly limited to only two, raising questions about the difficulty of compiling data related to core vaccine effectiveness outcomes.*

**Response:** The reviewer’s concern has been addressed in revised manuscript.

*8. The manuscript must follow proper scientific nomenclature. For example, after the first full mention of "Escherichia coli," subsequent references should use the abbreviated form "E. coli." The authors should check the entire text for this issue.*

**Response:** The reviewer’s concern has been addressed in revised manuscript

**Reviewer #2:**

*Dear Authors,*

*Your manuscript addresses a timely topic with valuable insights on APEC vaccines and machine learning applications. Clarification of meta-analysis methodology, data reporting, and the specific contribution of machine learning would strengthen rigor, transparency, and scientific impact.*

**Response:** We thank reviewers for taking time and constructively review our manuscript. The methodology has been updated with comprehensive and detailed explanation of quantitative synthesis (line #231 to 243) and qualitative synthesis (line # 219 to 230). The reporting of data extraction has been clarified in line # 199 to 212. The impact of ML insights has been clarified in methodology in Line #231 to 243 and in Table # 5. The forest plots and funnel plots have been updated and added from figure 4 to 7. The risk of bias assessment illustration is added as figure 2,3. Kindly review the revised version.
